# Relationship between Water Activity and Moisture Content in Floral Honey

**DOI:** 10.3390/foods8010030

**Published:** 2019-01-16

**Authors:** Chiachung Chen

**Affiliations:** Department of Bio-industrial Mechatronics Engineering, National Chung Hsing University, 250 Kuokuang Road, Taichung 40227, Taiwan; ccchen@dragon.nchu.edu.tw; Tel.: +886-4-22857562; Fax: +886-4-22857135

**Keywords:** honey, water activity, moisture content, regression, categorical testing

## Abstract

The water activity (Aw) and moisture content (MC) data of floral honey at five temperatures were determined using the Aw method and it was found that temperature significantly affected the Aw/MC data. The linear equation could be used to express the relationship between Aw and MC of honeys. The empirical regression equations between parameters and temperature were established. To evaluate the factors affecting the Aw/MC data, we used categorical tests of regression analysis to assess the effect of the correlation between Aw and MC of honey and examined the factors affecting the regression parameters. Six datasets from five countries were selected from the literature. The significance of the levels of qualitative categories was tested by *t*-test. The slope of the relationship between Aw and MC was affected by the state of honey (liquid and crystallized). The intercepts were significantly affected by honey type (flower or honeydew), harvesting year, geographical collection site, botanical source and other factors. The outliers in the datasets significantly affected the results. With modern regression analysis, useful information on the correlation between Aw and MC could be found. The results indicated that no universal linear equation for Aw and MC could be used. The Aw value could be used as a criterion for the honey industry; then, the MC of honey could be calculated by the specific linear equation between Aw and MC.

## 1. Introduction

The use of honey has a long history. The sweet food made by bees obtaining nectar from flowers is called flower honey. When bees obtain the sweet secretions of aphids or other insects, the product is called honeydew [1]. Honey is usually maintained in a liquid state. The other is called crystallized honey; many factors such as chemical composition, a degree of supersaturation, viscosity, a fructose/glucose ratio, moisture and dextrine content, water activity, micro-crystals and nucleation seeds presence, age, storage temperature and thermal history all influence its properties [2].

Fermentation is a problem for honey. The liquid mixture contains water, fructose and acid, so yeast could develop when the water content reaches a certain level [3]. The higher the water content, the greater the occurrence of fermentation and spoilage. The water content in called moisture content (MC) in the food industry. Rockland [4] determined that the amount of the free water is really a response to the development of yeast, and not MC. The amount of free water could be described as water activity (Aw). The Aw for honey ranges from 0.5~0.65 [5,6,7,8,9,10,11]. The limiting Aw for yeast in honey is about 0.61~0.62 [12] according to Beuchat et al. [12] and 0.60 [8,10,13,14] according to Beckh et al. [13], Gletier et al. [8], Ruegg and Blanc [14], and Zamora and Chirife [10].

The official method for MC measurement is refractometric measurement. It is inexpensive and easy to use. However, it cannot be directly used for crystallized honey. 

With a promising methods for measuring Aw, the MC of honey can then be calculated using a previously established empirical equation. Some experiments have been performed to determine the Aw and MC data of honey, subsequently establishing the relationship between Aw and MC by regression analysis. Several models have been proposed [5,6,7,9,11,13,14,15,16,17,18,19]. 

The factors affecting the Aw of honey included the type (flower or honey dew), state (liquid or crystallized), climatic and botanical origins, and induced fine granulation [5,6,10,19]. 

Glitter et al. [8] compared two types of honey: flower and honeydew, in liquid or crystallized state. At the same MC, Aw was higher for crystallized than for liquid honey. At the same Aw in liquid state, MC was higher for honeydew than for flower honey; there was no difference between the two types in the crystallized state. However, only correlation coefficients (r) between Aw and MC were reported in this study. No statistical methods were reported for comparison. 

Cavia et al. [6] compared three groups of honey from different climatic regions and harvest years. The *t*-test, used to assess differences between groups, revealed no significant difference among the groups. The author then pooled the three groups of datasets and proposed an empirical equation. In comparing this equation with that in the literature, the intercepts and slopes of these equations differed. The authors mention these differences related to the measurement of Aw and MC. 

Chirife et al. [7] established a linear equation for Aw and MC from 36 liquid Argentinian honey samples and compared this equation with that from Beck et al. [13]. The slope and intercept differed, and the results were attributed to the lack of accuracy of measurement techniques and the inference of different botanical sources and geographical collection sites. Abramovic et al. [5] investigated the correlation between Aw and MC for flower and honeydew honey in Slovenia. At the same MC, Aw was higher for honeydew than for flower honeys. The two datasets were pooled and a linear equation for Aw and MC was established. Comparing regression parameters with five other empirical equations in the literature, the difference found was attributed to the sugar composition and measurement of Aw. Zamora et al. [11] examined the data from Beckh et al. [13] in fluid, partially crystalline and crystalline honey and found no significant differences by F-test. However, the determination of coefficient $R^{2}$ was only 0.53 for the pooled data. Zamora et al. [11] found no significant difference in the relationship between Aw and MC for honey by botanical source or collection site. Comparing four regression equations in the literature, the authors found that the slope for the three datasets was similar [11]. Perez et al. [8] reviewed 10 datasets for Aw and MC in flower honey and proposed a weighted average regression equation by using meta-analysis.

Recently, modern regression analysis was proposed to analyze treatment [20,21,22]. If the influencing factor for the treatment has several levels of qualitative categories, the significance of these treatments can be tested by categorical testing with regression analysis [20,21,22].

The objectives of this study were to: (1) determine the Aw/MC relationships for floral honey between 10 and 30 °C; and (2) evaluate the factors affecting the sorption isotherms of honey with previously published data by using regression analysis.

## 2. Materials and Methods 

### 2.1. Materials 

The floral honey used for this study was Longyuan honey collected at the Chunglaun Township, Nantou, Taiwan. The initial moisture content of the sample was 17.16% on a w.b.

The desired moisture content for storage and processing ranges from 17% to 22% w.b. The samples were rewetted by adding the amount of the water necessary to reach the desired moisture content. The sample preparation was performed according to the study by Shen and Chen [23]. All samples were sealed in glass vessels and stored at 5 °C for three weeks to ensure uniform moisture content. The moisture content of the honey was measured by using the ATAGO DR-A1 ABBE Refractometer (Atago Inc., Bellevue, WA, USA). The total number of sample in this study was 27. There were nine moisture contents, and three replicates of each moisture content. 

### 2.2. RH Meter

The THT-V2 humidity transmitter (Shinyei technology, Kobe, Japan) was used to determine the water activity of the floral honey. These RH sensors were calibrated with several saturated salt solutions, and the accuracy of the RH meter was within 0.7% RH after calibration. All measured RH values were transformed into actual values by using previously established calibration equations to enhance its accuracy.

### 2.3. Aw Method

**Figure 1:**
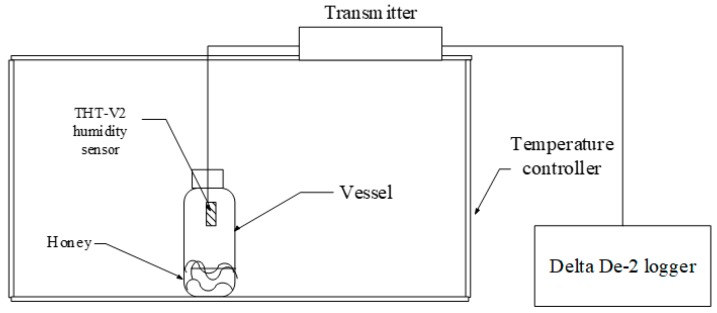
Diagram of the experimental set-up.

The set-up for the Aw method is shown in Figure 1. Samples at known moisture contents were placed in a glass vessel, sealed with a rubber stopper to ensure airtight conditions, and then placed in a temperature-controlled chamber that was maintained at 5 °C. The volume of the vessel was 250 mL. When the temperature and the RH within the container were stabilized, the vapor pressure of the samples and the interstitial air in the vessel reached the equilibrium state. The RH and temperature values were determined. To ensure the equilibrium state, each temperature level was maintained for 12 h, then adjusted to next temperature level. All Aw values were measured at five temperatures (10, 15, 20, 25 and 30 °C). After finishing the experiments, the samples were taken from each vessel to determine the moisture content using the ATAGO DR-A1 ABBE Refractometer.

This technique has been used to determinate sorption isotherm for autoclaved aerated concrete [24], sweet potato slices [25], pea seeds [26] and Oolong tea [27].

### 2.4. Literature Survey 

The six datasets from five countries used to evaluate the factors affecting the regression parameters between Aw and MC are in Table 1. The published models used, along with the data from the literature and seven other published models, are displayed in Table 2.

### 2.5. Categorical Tests

If the influencing factor has several levels of qualitative categories, the significance of the qualitative treatment could be tested by *t*-test or F-test.

#### 2.5.1. Testing the Slope for Two Treatments

To evaluate the effect of categorical variables such as type or state of honey, an indicator variable is used. The equation for the regression line relating two types of datasets that differ in both intercept and slope are as follows:

(1) $$y=b_{o}+b_{1}X_{1}+b_{2}Z_{1}+b_{3}X_{1}Z_{1}+\varepsilon$$

$$Z_{1}=0,if\;the\;data\;is\;from\;f\mathrm{actor}\;A,$$

$$\;\;Z_{1}=1,if\;the\;data\;is\;from\;f\mathrm{actor}\;B$$

(2) $$\mathrm{For}\;\mathrm{factor}\;A:\;y=b_{o}+b_{1}X_{1}+\varepsilon$$

(3) $$\mathrm{For}\;\mathrm{factor}\;A:\;y=\left(b_{o}+b_{2}\right)+\left(b_{1}+b_{3}\right)X_{1}+\varepsilon$$

$$H_{0}:b_{2}=b_{3}=0$$

$$H_{1}:b_{2}\neq 0,b_{3}\neq 0$$

To test the hypothesis that two regression lines have the same slope or intercept, we could use the *t*-test.

#### 2.5.2. Testing the Slope for Three Treatments

For three treatments, the regression equations relating datasets that differ in both intercept and slope are as follows:

(4) $$\;y=b_{o}+b_{1}X_{1}+b_{2}Z_{1}+b_{3}Z_{2}+b_{4}X_{1}Z_{1}+b_{5}X_{1}Z_{2}+\varepsilon$$

$$Z_{1}=Z_{2}=0,if\;the\;observation\;is\;from\;A,$$

$$Z_{1}=1\;and\;Z_{2}=0,if\;the\;observation\;is\;from\;B,$$

$$Z_{1}=0\;and\;Z_{2}=1,if\;the\;observationis\;from\;C,$$

(5) $$\mathrm{For}\;\mathrm{factor}\;A:\;y=b_{o}+b_{1}X_{1}+\varepsilon$$

(6) $$\mathrm{For}\;\mathrm{factor}\;B:\;y=\left(b_{o}+b_{2}\right)+\left(b_{1}+b_{4}\right)X_{1}+\varepsilon$$

(7) $$\mathrm{For}\;\mathrm{factor}\;C:\;y=\left(b_{o}+b_{3}\right)+\left(b_{1}+b_{5}\right)X_{1}+\varepsilon$$

$$H_{0}:b_{4}=b_{5}=0$$

$$H_{1}:b_{4}\neq 0,b_{5}\neq 0$$

#### 2.5.3. Two Indicator Variables

If the qualitative variables have two qualitative factors (e.g., flower and honeydew, crystallized and liquefied), the regression line can be expressed as follows:

(8) $$y=b_{o}+b_{1}X_{1}+b_{2}Z_{1}+b_{3}Z_{2}+b_{4}X_{1}+b_{5}X_{1}Z_{2}+\varepsilon$$

$$a:\;\mathrm{liquefied},\;\mathrm{flower}:\;Z_{1}=0,Z_{2}=0$$

(9) $$y=b_{o}+b_{1}X_{1}+\;\varepsilon$$

$$b:\;\mathrm{crystallized},\;\mathrm{flower}:\;Z_{1}=0,Z_{2}=1$$

(10) $$y=\left(b_{o}+b_{3}\right)+\left(b_{o}+b_{5}\right)X_{1}+\varepsilon$$

$$c:\;\mathrm{liquefied},\;\mathrm{honeydew}:\;Z_{1}=1,Z_{2}=0$$

(11) $$y=\left(b_{o}+b_{2}\right)+\left(b_{o}+b_{4}\right)X_{1}+\varepsilon$$

$$d:\;\mathrm{crystallized},\;\mathrm{honeydew}:\;Z_{1}=1,Z_{2}=1$$

(12) $$y=\left(b_{o}+b_{2}+b_{3}\right)+\left(b_{1}+b_{4}+b_{5}\right)X_{1}+\varepsilon$$

## 3. Results

### 3.1. Water Activity of Honey

**Figure 2:**
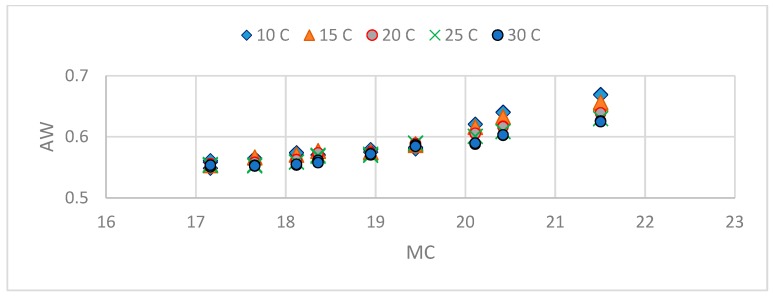
Effect of temperature on the water activity (Aw)/moisture content (MC) data of floral honey.

The Aw/MC data at five temperatures are shown in Figure 2. Temperature significantly affected the Aw/MC data. 

**Figure 3:**
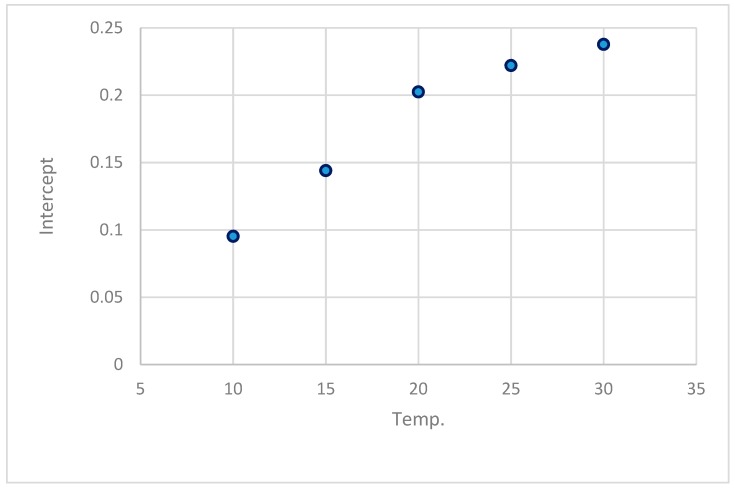
Effect of temperature on parameter A (intercept) of the linear equation.

**Figure 4:**
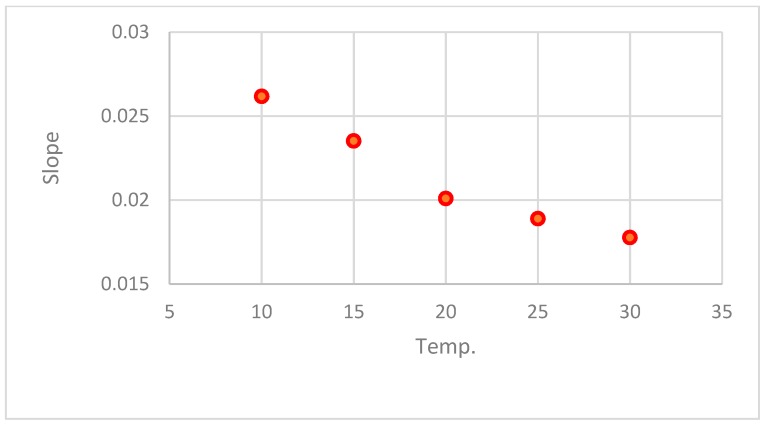
Effect of temperature on parameter B (slope) of the linear equation.

The results of the estimated parameters and comparison statistics for the linear equation at different temperature are in Table 3. The effect of temperature on parameters A and B is shown in Figure 3 and Figure 4. 

The empirical regression equations between parameters and temperature were established. The equation for A and B was expressed as:

(13) $$A\;=\;-0.06999\;-\;0.0019264\mathrm{Temp}\;+\;3.000030\;\times 10^{-4}{\mathrm{Temp}}^{2},R^{2}=0.991$$

(14) $$B\;=\;0.03516\;-\;0.0010345\mathrm{Temp}\;+\;1.4149\;\times 10^{-5}{\mathrm{Temp}}^{2},R^{2}=0.990$$

Three forms of the linear equation that incorporated the temperature term were proposed as follows:

(15) $$\mathrm{Aw}\;=\;-0.06999\;-\;0.0019264\mathrm{Temp}\;+\;3.000030\;\times 10^{-4}{\mathrm{Temp}}^{2}\;+\;0.3516\;-0.0010345\mathrm{Temp}\;+\;1.4149\times 10^{-5}{\mathrm{Temp}}^{2})\mathrm{MC}$$

### 3.2. Comparison with Published Data

**Figure 5:**
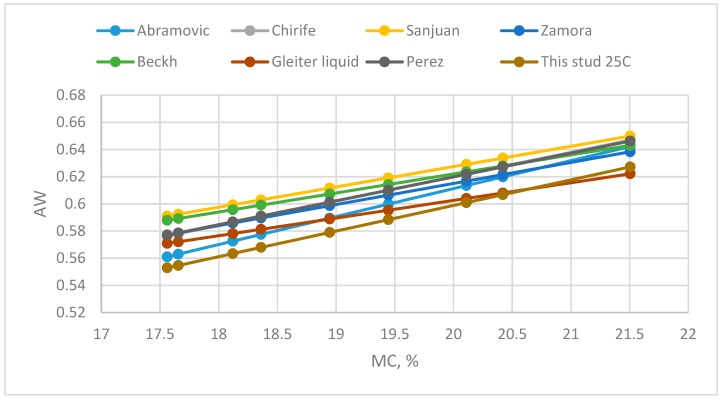
Comparison of Aw/MC equation at 25 °C with models from literature: [5,7,8,9,10,12,18].

The Aw/MC linear equation of floral honey at 25 °C in this study was compared with published data (Figure 5). At MC < 20.5%, the Aw values of this study were lower than other data. However, when MC > 20.5%, the Aw values of this study were higher than those of Gleiter et al. [8]. The reason for this could be that the Aw/MC data were affected by honey type (flower or honeydew), harvesting year, geographical collection site, botanical source and other factors. Further study was executed to study the factors influencing the Aw/MC data.

### 3.3. Effect of Honey Type on Aw 

**Figure 6:**
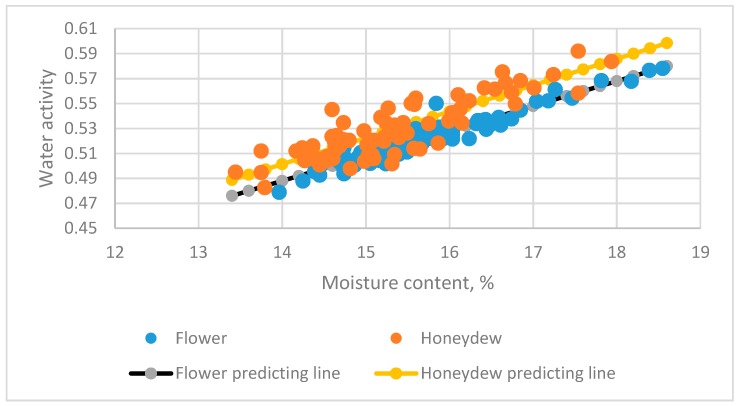
The relationship between water activity (Aw) and moisture content (MC) of flower and honeydew honey in Slovenia [5].

Two types of honey (flower and honeydew) [5] were used to evaluate the factors affecting the relationship between Aw and MC by Equation (1). The data distribution and predicted lines are in Figure 6. 

The results of the linear regression are as follows:

(16) $$\begin{array}{ccccc} \mathrm{Aw}= & 0.20801+ & 0.019985\mathrm{MC}- & 0.00189Z+ & 0.00108Z\cdot \mathrm{MC}\\ & \left(11.45\right) & \left(17.29\right) & \left(-0.074\right) & \left(0.66\right) \end{array}$$

$$R^{2}\;=\;0.823$$

where *Z* is the categorized variable, *Z* = 0 is flower honey and *Z* = 1 is honeydew honey. The numbers in parentheses below the estimated values of parameters are *t*-test values for the estimated value. The *t* and *p* values for Z·MC were 0.665 and 0.112, respectively. Type had no significant effect on the Aw and MC relationship.

The adequate equation was as follows:

(17) $$\begin{array}{cccc} \mathrm{Aw}= & 0.19964+ & 0.020579\mathrm{MC}+ & 0.014766Z\\ & \left(15.49\right) & \left(25.06\right) & \left(9.18\right) \end{array}$$

For flower honey,

Aw = 0.19964 + 0.020579MC(18)

For honeydew honey,

Aw = 0.18171 + 0.020579MC(19)

From Equation (17)–(19), we found no significant difference in the slope of the linear equation for flower and honeydew honey. However, the intercept significantly differed with two types of honey.

### 3.4. Effect of the Type and State of Honey on the Aw and MC Relationship

The datasets for Glitter et al. [8] included different honey types (flower and honeydew) and states (liquid and crystallized). Two indicator variables were analyzed by Equation (8). 

The regression equation was as follows:

(20) $$\begin{array}{cccccc} \mathrm{Aw}= & 0.30845+ & 0.016905\mathrm{MC}+ & 0.018156Z_{1}+ & 0.034193Z_{2}- & 0.00391Z_{2}\cdot \mathrm{MC}\\ & \left(24.12\right) & \left(21.83\right) & \left(14.76\right) & \left(2.71\right) & \left(-3.93\right) \end{array}$$

$$R^{2}\;=\;0.831$$

For crystallized flower honey, $Z_{1}$ = 0, $Z_{2}$ = 0,

Aw = 0.340845 + 0.016905MC(21)

For liquid flower honey, $Z_{1}$ = 0, $\;Z_{2}$ = 1.0

Aw = 0.34264 + 0.012995MC(22)

For crystallized honeydew honey,$\;Z_{1}=1.0,\;\;Z_{2}=0$

Aw = 0.32661 + 0.016905MC(23)

For liquid honeydew honey, $Z_{1}=1.0,\;\;Z_{2}=1.0$

Aw = 0.36080 + 0.012995MC(24)

**Figure 7:**
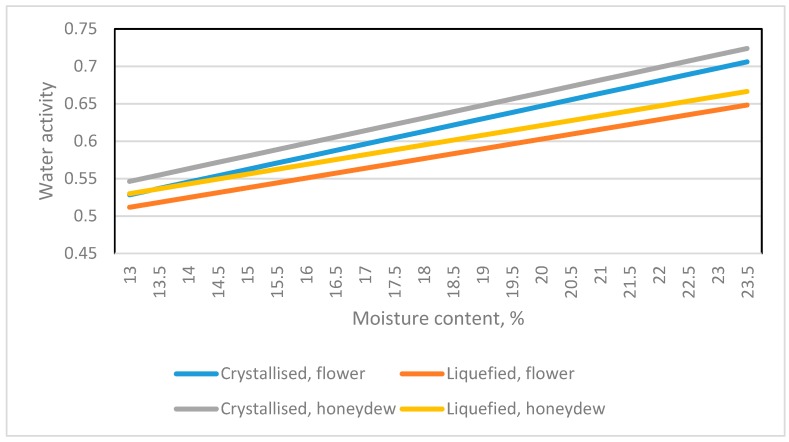
The prediction equations between water activity and moisture content including different type (flower and honeydew) and state (liquid and crystallized) of honey [8].

The results indicated a significant difference in the intercept. With the same crystallized state, flower and honeydew honey had a similar slope, 0.016905. With the same liquid state, the slope was 0.012995. That is, the state not the type of honey significantly affects the slope parameter of the Aw linear equation. The prediction lines of two states and two types of honey are in Figure 7.

### 3.5. Comparison of the Correlation between Aw and MC with Two Datasets

Several datasets for Aw and MC for honey were used to evaluate factors affecting correlation between the Aw and MC.

#### 3.5.1. Argentinian [7] and Slovenian Honeys [5]

**Figure 8:**
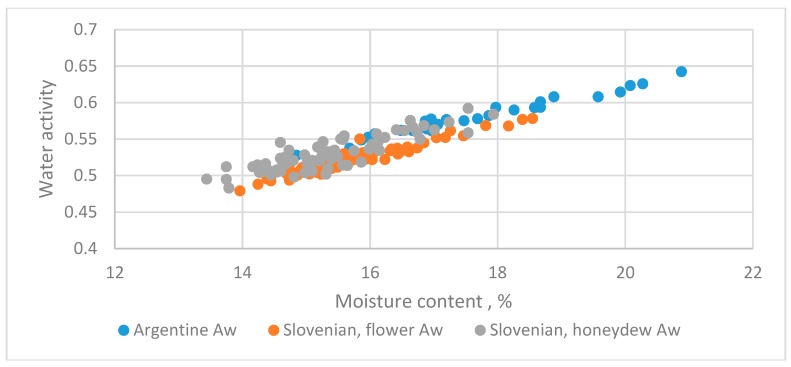
The relationship between water activity and moisture content of flower and honeydew honey from Argentinian [7] and Slovenia [5].

The datasets from different countries with the liquid state are in Figure 8. 

Two datasets for Slovenia honey were pooled and evaluated by Equation (4). The regression equation was as follows:

(25) $$\begin{array}{ccccc} \mathrm{Aw}= & 0.22826+ & 0.019149\mathrm{MC}+ & 0.0412Z- & 0.00147Z\cdot \mathrm{MC}\\ & \left(15.64\right) & \left(20.39\right) & \left(1.449\right) & \left(-0.901\right) \end{array}$$

*R*^2^ = 0.872

The *t*-test value for *Z*·MC was −0.901 and not significant. The adequate linear equation was as follows:

(26) $$\begin{array}{cccc} \mathrm{Aw}= & 0.23581+ & 0.018662\mathrm{MC}+ & 0.015256Z\\ & \left(19.75\right) & \left(24.30\right) & \left(5.66\right) \end{array}$$

*R*^2^ = 0.873

#### 3.5.2. German and Slovenian Honeys

The datasets for Aw and MC for the two countries had the same slope, but a different intercept.

**Figure 9:**
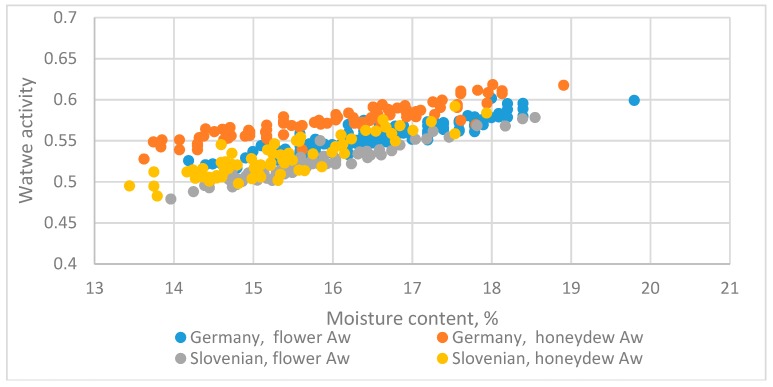
The relationship between water activity and moisture content for datasets for honey from Germany (pooled of the flower and honeydew, liquid state) [8] and Slovenia (liquid state) [5].

The datasets from Germany (pooled flower and honeydew, liquid state) [8] and Slovenia (liquid state) honey [5] are in Figure 9 and were used for assessing the influencing factors. 

The linear equation was as follows:

(27) $$\begin{array}{ccccc} \mathrm{Aw}= & 0.22826+ & 0.019149\mathrm{MC}+ & 0.095601Z- & 0.00302Z\cdot \mathrm{MC}\\ & \left(14.40\right) & \left(18.76\right) & \left(4.88\right) & \left(-1.080\right) \end{array}$$

*R*^2^ = 0.895

The *t*-test value for Z·MC was insignificant. The adequate equation is as follows:

(28) $$\begin{array}{cccc} \mathrm{Aw}= & 0.31305+ & 0.013680\mathrm{MC}+ & 0.026313Z\\ & \left(28.94\right) & \left(19.74\right) & \left(15.31\right) \end{array}$$

*R*^2^ = 0.893

The type (flower or honeydew) did not significantly affect the slope.

#### 3.5.3. Mixed-Source and Slovenian Honeys

**Figure 10:**
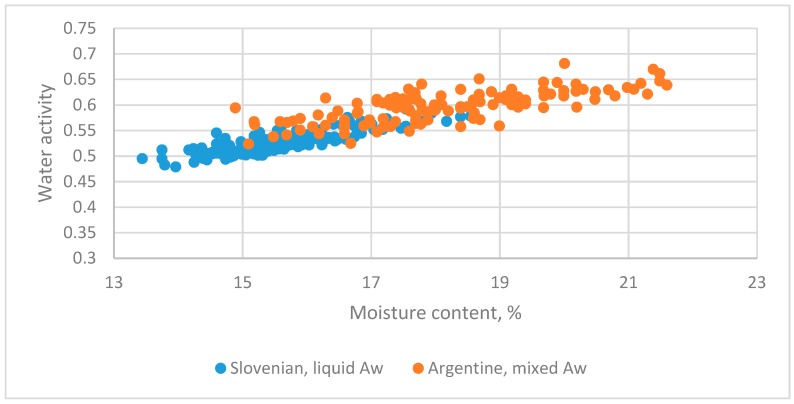
The relationship between water activity and moisture content for datasets for honey of different types and states [13] and Slovenia (pooled data of liquid states: flower and honeydew) [5].

The datasets from different types and states [13] and Slovenian honey (pooled liquid states: flower and honeydew) [5] are evaluate in Figure 10. 

The linear regression was as follows:

(29) $$\begin{array}{ccccc} \mathrm{Aw}= & 0.22826+ & 0.019149M+ & 0.11401Z- & 0.00507Z\cdot \mathrm{MC}\\ & \left(13.31\right) & \left(13.43\right) & \left(4.0513\right) & \left(-2.96\right) \end{array}$$

*R*^2^ = 0.861

The *Z*·MC had a *t*-test value of -2.96 and *p* = 0.00336, showing a significant effect. Both datasets had different slopes and intercepts.

Chirife et al. [7] compared the correlation for Argentina fluid honey and mixed honey from different countries [13] and found that the intercept and slopes of both differed. These results could be explained by the source of the honey. The Argentinian honey was liquid, the mixed honey included liquid, crystallized and partially crystallized states.

#### 3.5.4. Spanish and Slovenian Honeys

**Figure 11:**
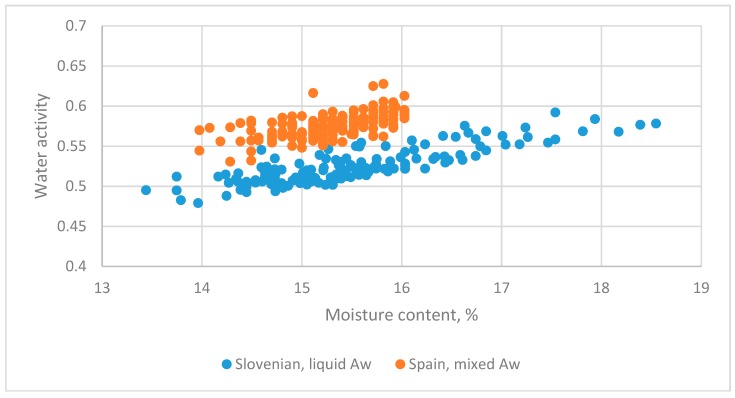
The relationship between water activity and moisture content for datasets for honey from Spain (flower honeys, unknown state) [6] and Slovenia (pooled data of liquid states, flower and honeydew) [5].

The datasets from Spain (flower honey, unknown state) [6] and Slovenia (pooled data of liquid states, flower and honeydew) [5] are in Figure 11. The regression equation was as follows:

(30) $$\begin{array}{ccccc} \mathrm{Aw}= & 0.22826+ & 0.019149\mathrm{MC}+ & 0.094645Z- & 0.00273Z\cdot \mathrm{MC}\\ & \left(13.41\right) & \left(17.48\right) & \left(5.03\right) & \left(-2.30\right) \end{array}$$

*R*^2^ = 0.899

The *t*-test value for *Z*·MC was −2.30, with *p* = 0.022, so datasets for the two countries had different slopes and intercepts.

#### 3.5.5. German (Crystallized State) and Slovenian (Liquid State) Honeys

**Figure 12:**
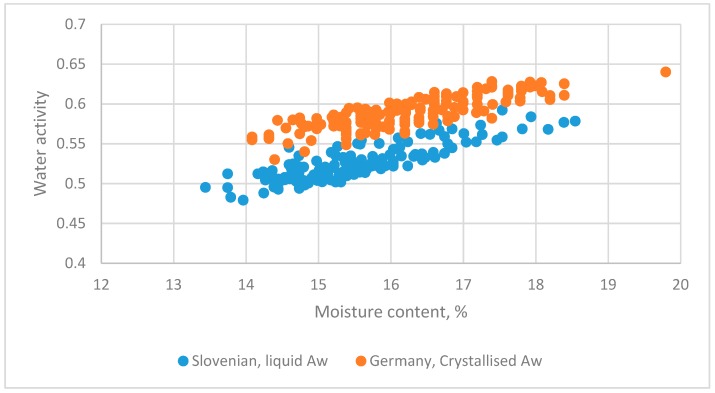
The relationship between water activity and moisture content for datasets for honey from Germany [8] and Slovenia [5].

The datasets from Gleiter et al. [8] and Abramovic et al. [5] for honey in different states are assessed (Figure 12). 

The regression equation was as follows:

(31) $$\begin{array}{ccccc} \mathrm{Aw}= & 0.22826+ & 0.019149\mathrm{MC}+ & 0.14899Z- & 0.00719Z\cdot \mathrm{MC}\\ & \left(12.01\right) & \left(15.66\right) & \left(6.46\right) & \left(-5.34\right) \end{array}$$

*R*^2^ = 0.746

The linear equations for the two datasets had different slopes and intercepts.

#### 3.5.6. Comparing the Correlation between Aw and MC with Three Datasets

**a. Case 1**

Three datasets were used: German liquid flower ($Z_{1}$ = 0, $Z_{2}=0$) and honeydew ($Z_{1}$ = 1,$\;Z_{2}=0$) honey [8] and Argentinian liquid honey ($Z_{1}$ = 0,$\;Z_{2}=1$) [7].

The linear equation was as follows:

(32) $$\begin{array}{ccccc} \mathrm{Aw}= & 0.31565+ & 0.014452\mathrm{MC}+ & 0.009275Z_{1}+ & 0.02677Z_{2}\\ & \left(39.61\right) & \left(30.26\right) & \left(4.87\right) & \left(20.06\right) \end{array}$$

*R*^2^ = 0.834

The slope of the three datasets was identical, and the intercepts significantly differed.

**b. Case 2**

Three datasets, Slovenian [5] liquid honeydew ($Z_{1}$ = 0,$\;Z_{2}=0$) and flower honey ($Z_{1}\;$= 1,$\;Z_{2}=0$) and Argentinian liquid honey [7], were used.

The linear equation was as follows:

(33) $$\begin{array}{ccccc} \mathrm{Aw}= & 0.21471+ & 0.019558\mathrm{MC}+ & 0.020636Z_{1}+ & 0.014421Z_{2}\\ & \left(21.62\right) & \left(31.07\right) & \left(9.15\right) & \left(9.54\right) \end{array}$$

*R*^2^ = 0.917

The slope of the three datasets was identical and the intercepts significantly differed.

Zamora et al. [10] compared regression equations for Aw and MC for honey from different countries and found no significant difference based on botanical source or geographical collection site. Our study confirms these results.

**c. Case 3**

Three datasets, German crystallized flower and honeydew honey ($Z_{1}$ = 0,$\;Z_{2}=0$) [8], Argentinian liquid honey ($Z_{1\;}$= 1,$\;Z_{2}=0$) [7] and Slovenian liquid honeydew and flower honey ($Z_{1}$ = 0,$\;Z_{2}=1$) [5], were used. The linear equation was as follows:

(34) $$\begin{array}{ccccccc} \mathrm{Aw}= & 0.31724+ & 0.01136\mathrm{MC}- & 0.14899Z_{1}- & 0.10886Z_{2}+ & 0.007789Z_{1}\cdot \mathrm{MC}+ & 0.006316Z_{2}\cdot \mathrm{MC}\\ & \left(29.95\right) & \left(14.77\right) & \left(-6.70\right) & \left(-3.38\right) & \left(5.53\right) & \left(3.42\right) \end{array}$$

*R*^2^ = 0.782

The slopes and intercepts of the three datasets significantly differed.

#### 3.5.7. Outlier Detection

The intercept and slope of the equation for the dataset of flower honey from La Palma Island, Spain, significantly differed from those in other datasets [9]. Outlier data (17.2233, 0.6084) was found by the Cook’s distance test [21]. The original linear equation proposed by the authors was as follows:

(35) $$\mathrm{Aw}\;=\;0.35732\;+\;0.01349\mathrm{MC},\;R^{2}\;=\;0.63$$

After deleting this data, the new equation was as follows:

(36) $$\mathrm{Aw}\;=\;0.32842\;+\;0.01495\mathrm{MC},\;\;R^{2}\;=\;0.93$$

**Figure 13:**
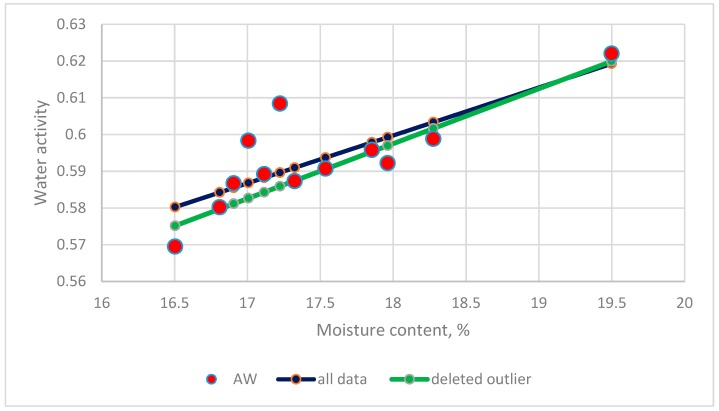
The comparison between two equations with and without outliers in the datasets of Sanjuan et al. [9].

The comparison between Equations (32) and (33) is shown in Figure 13.

After deleting this data point, the slope, intercept and coefficient of determination changed obviously. 

If we compare all data from Sanjuan et al. [9] with the datasets for Argentinian honey [7], the linear equation was as follows:

(37) $$\begin{array}{ccccc} \mathrm{Aw}= & 0.26838+ & 0.017675\mathrm{MC}- & 11.4662Z+ & 48.35758Z\cdot \mathrm{MC}\\ & \left(0.48\right) & \left(0.59\right) & \left(-3.22\right) & \left(8.15\right) \end{array}$$

*R*^2^ = 0.960

The intercept and slope for the two datasets differed significantly. 

If the outlier was deleted from the datasets of Sanjuan et al. [9], the equation for evaluating the two datasets was as follows:

(38) $$\begin{array}{cccc} \mathrm{Aw}= & 0.27478+ & 0.017310\mathrm{MC}+ & 0.013714Z\\ & \left(25.40\right) & \left(28.19\right) & \left(7.31\right) \end{array}$$

*R*^2^ = 0.956

The slope of the two datasets was identical. From the results of Equations (37) and (38), the outlier significantly affected the comparison results for the two datasets. With modern regression analysis, more useful information on correlation between Aw and MC could be found. The results indicated the importance of finding the correct equation with modern regression.

## 4. Discussion

Based on the study of the datasets of Gleiter et al. [8], the intercept parameters differed significantly. The slope parameter could be classified into two categories: liquid and crystallized. Thus, the slope for the linear equation was affected only by the state of the honey. The type of honey, flower and honeydew, and other factors did not affect the slope but did affect the intercept. 

Chirife et al. [7] found that Aw in honey was determined mainly by the concentrations of fructose and glucose that are most abundant in honey. The authors developed an Aw equation from the effect of the osmotic concertation on the osmotic coefficient, which was as follows:

Aw = Exp(−Φ0.018mv)(39)

where Φ is the osmotic coefficient, m is molality and v is the number of moles of kinetic units.

By Taylor’s expansion, and assuming 0.018mv << 1, the new relationship is as follows:

Aw = 1 − Km(40)

For very concentrated and small intervals of sugar solutions, Equation (40) was rewritten as follows:

(41) $$\mathrm{Aw}\;=\;A\;-\;B\;(s)\;=\;b_{0}+b_{1}\mathrm{MC}$$

where (s) is the solid concentration in water, and A and B are constants.

In this study, we found the slope to be affected only by the state of the honey (liquid or crystallized). The other factors, such as type (flower or honeydew), geographical collection sites and botanical source did not significantly affect the slope, but did affect the intercept of the Aw equation. 

Perez et al. [18] selected 10 datasets of flower honey to study the relationship between Aw and MC and found similar but not identical the slopes and intercepts of these linear regressions. The authors attributed the finding to sampling error, accuracy of the Aw measurement, and variation in sugar composition. The slopes for 10 datasets ranged from 0.0149 to 0.0197. However, the state of honey was not mentioned in this research. We found a slope of 0.016905 for crystallized honey and 0.012995 for liquid honey for the datasets of Gleiter et al. [8]. The wide slope range for the 10 datasets from the study of Perez et al. [18] may be explained by the effects due to the state of honey. The slope for the linear equation for Slovenia honey [5] and five other datasets ranged from 0.014 to 0.0196. The difference in parameters was attributed to sugar composition and the Aw determination methods by the authors. The significant difference between the two maximum and minimum slope values, 0.014 and 0.0196, could be explained by the state of the honey. 

The study by Cavia et al. [6] included three groups of samples. The *G*_1_ and *G*_2_ datasets were obtained in 1996 and 1998 from a continental climate, and the *G*_3_ datasets was obtained in 1998 from an oceanic climate. The slopes for the Aw model were 0.02149 and 0.02362 for *G*_1_ and *G*_2_ and 0.01476 for *G*_3_. The significant difference between the three slopes could be explained by the effect of climate on the state of the honey. The crystallized state enhanced by the continental climate may be due to the difference in slope values.

The MC of honey is considered to be the criterion for the honey industry. The MC is usually determined by the refractometric technique. The method is simple and inexpensive. However, the MC could be affected by weather conditions, original moisture content of the nectar, and environmental temperature and humidity after harvesting. The storage materials and sealed technique also affect MC. The MC of the crystallized state cannot be directly measured by refractometer.

The Aw is measured by some commercial equipment. The criterion of Aw < 0.6 may be used as a safety standard to prevent the development of osmotolerant yeasts. Recently, the performance of electronic hygrometers has been improved. They have been used to determine the Aw of tea leaves and other materials [23,24].

In this study, we found an effect of factors on slope for the correlation between Aw and MC. The state of honey, crystallized and liquid, had a significant effect on the slope value. However, other factors, such as harvesting year, botanic source and collection sites did not affect the slope but did affect the intercept. Therefore, no universal linear equation for Aw and MC could be established. The Aw value may be used as the criterion for the honey industry and directly determined by an electronic hygrometer. Then the MC of honey could be calculated by the specific linear equation between Aw and MC. The effect of the temperature needs to be considered. 

In the traditional MC and AW determination method, honey must be liquefied previously, so that all crystals are totally melted, such that all measurements can be done with liquid honey. By the Aw method used in this study, the Aw values of crystallized honey could be determined directly in the crystalline state. 

In this study, the moisture content of liquid honey was measured by using a refractometer. There are two official procedures Association Official Analytical Chemists (AOAC) and European Honey Commission (EHC) [28,29] for determining the moisture content. A comparison between the official method and the refractometer has been reported [30]. The comparison between the official method and the refractometer of floral honey used in this study will be further studied.

## 5. Conclusions

Conclusions were drawn from the results of this study.

The Aw/MC data at five temperatures were determined, and temperature significantly affected the Aw/MC data. The linear equation could be used to express the relationship between Aw and MC of Honeys. The empirical regression equations between parameters and temperature were established. The intercept and slope of the linear equation could be expressed as the polynomial equations.

The slope of the correlation between Aw and MC was affected by the state of honey (liquid and crystallized). The intercept was significantly affected by honey type (flower or honeydew), harvesting year, geographical collection site and botanical source. The outliers in the dataset significantly affected the comparison results. Modern regression analysis can provide useful information for the correlation between Aw and MC. No universal linear equation for Aw and MC could be established. The Aw value may be used as the criterion for honey industry, and then the MC of honeys can be calculated by the specific linear equation between Aw and MC. 

## Figures and Tables

**Table 1:** Selected studies on the relationship between water activity and moisture content in honey.

Types	Geographical Original of Honeys	Aw Determination Method	Sample Size	Moisture Range (%)	Reference
Honeydew and flower	Slovenia	Cx-2T Chill-mirror Aw system	150	13.4-18.6	Abramovic et al. [5]
Flower	Spain (liquid and crystallized)	Cx-2T Chill-mirror Aw meter	90	14.2-21.5	Cavia et al. [6]
Flower	Argentine	Aqual series 3 Model TE dew-point Aw meter	35	13.8-20.8	Chirife et al. [7]
Honeydew and flower	Germany	Navasian Aw meter	166	14.2-22.7	Gleiter et al. [8]
Flower	Spain	Aqual series 3 Model TE dew-point Aw meter	13	16.5~19.4	Sanjuan et al. [9]
Flower	Argentine (liquid and crystallized)	Aqual series 3 Model TE dew-point Aw meter	36	15.8~27.1	Zamora et al. [11]

**Table 2:** Published equations of water activity and moisture content of honey.

Study	Equations	$R^{2}\;(r)$	Reference
I. Datasets used in this study.			
1.	Aw = 0.23 + 0.019MC	(0.843)	Abramovic et al. [5]
2.	Aw = 0.2674 + 0.01955MC	(0.709)	Cavia et al. [6]
3.	Aw = 0.262 + 0.0179MC	0.969	Chirife et al. [7]
4.	Aw = 0.35732 + 0.01349MC	0.654	Sanjuan et al. [9]
5.	Aw = 0.305 + 0.0155MC	0.969	Zamora et al. [11]
II. Datasets not used in this study.			
1.	Aw = 0.13 + 0.025MC	(0.8230)	Alcala and Gomez [15]
2.	Aw = 0.342 + 0.014MC	(0.723)	Beckh et al. [13]
3.	Aw = 0.25643 + 0.01965MC	(0.813)	Estupinan et al. [16]
4.	Aw = 0.38242 + 0.01211MC	(0.765)	Millan et al. [17]
5.	Aw = 0.2686 + 0.01756MC	Meta-analysis	Perez et al. [18]
6.	Aw = 0.0.271 + 0.0177MC	(0.901)	Ruegg and Blanc [14]
7.	Aw = 0.248 + 0.0175MC	(0.973)	Salamanca et al. [19]

**Table 3:** Estimated values of parameters in the linear equation.

Temperature °C	Parameters		$\mathrm{Coefficients}\;\mathrm{of}\;\mathrm{Determination}\;R^{2}$	Standard of Deviations of Estimated Values s
	A	B		
10	0.09520	0.026172	0.908	0.3195
15	0.14395	0.023511	0.936	0.2611
20	0.20243	0.020089	0.987	0.2165
25	0.22204	0.018839	0.968	0.1710
30	0.23771	0.017768	0.958	0.1536
